# Physical Chemistry Education and Research in an Open-Sourced Future

**DOI:** 10.1021/acsphyschemau.3c00078

**Published:** 2024-04-01

**Authors:** Jeffrey T. DuBose, Soren. B Scott, Benjamin Moss

**Affiliations:** † Division of Chemistry and Chemical Engineering, California Institute of Technology, Pasadena, California 91125, United States; ‡ Department of Chemistry, University of Copenhagen, Universitetsparken 5, Copenhagen 2100, Denmark; § Department of Materials & iX institute, Imperial College London Exhibition Rd, South Kensington, London SW7 2BX, United Kingdom

**Keywords:** technical education, physical chemistry education, open source, inclusion, mentoring

## Abstract

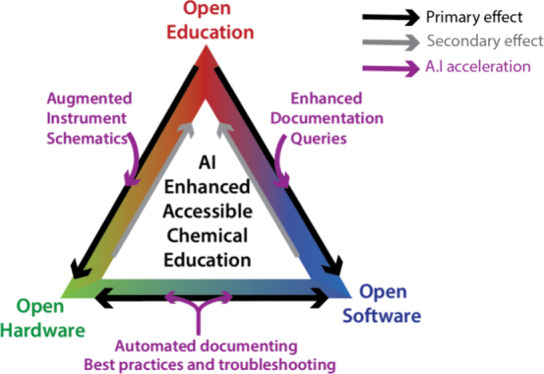

Proficiency in physical chemistry requires a broad skill set. Successful trainees often receive mentoring from senior colleagues (research advisors, postdocs, etc.). Mentoring introduces trainees to experimental design, instrumental setup, and complex data interpretation. In lab settings, trainees typically learn by customizing experimental setups, and developing new ways of analyzing data. Learning alongside experts strengthens these fundamentals, and places a focus on the clear communication of research problems. However, this level of input is not scalable, nor can it easily be shared with all researchers or students, particularly those that face socioeconomic barriers to accessing mentoring. New approaches to training will therefore progress the field of physical chemistry. Technology is disrupting and democratising scientific education and research. The emergence of free online courses and video resources enables students to learn in a style that suits them. Higher degrees of automation remove cumbersome and sometimes arbitrary technical barriers to learning new techniques, allowing one to collect high quality data quickly. Open sourcing of data and analysis tools has increased transparency, lowered barriers to access, and accelerated scientific dissemination. However, these advances also can lead to “black box” approaches to acquiring and analyzing data, where convenience replaces understanding and errors and misrepresentations become more common. The risk is a breakdown in education: *if one does not understand the fundamentals of a technique or analysis, it is difficult to correctly discern the practical limits of an experiment, distinguish signal from noise, troubleshoot problems, or take full advantage of powerful analytical procedures*. Our vision of the future of physical chemistry is built around democratized learning, where deep technical and analytical expertise from physical chemists is made freely available. Advancements in technical education through expert-generated educational resources and AI-based tools will enrich physical chemistry education. A holistic approach to education will prepare the physical chemists of 2050 to adapt to rapidly advancing technological tools, which accelerate the pace of research. Technical education will be enhanced by accessible open-source instrumentation and analysis procedures, which will provide instruments and analysis scripts specifically designed for education. High quality, comparable data from standardized open-source instruments will feed into accessible databases and analysis projects, providing others the opportunity to store and analyze both failed and successful experiments. The coupling of open-source education, hardware, and analysis will democratize physical chemistry while addressing risks associated with “black box” approaches.

## Introduction

Physical chemists must possess a diverse skill set to probe the fundamental underpinnings of chemical systems. A physical chemist might, for example, be adept in the accurate measurement and modeling of in situ spectroscopic, electrochemical, and thermodynamic/kinetic data, while also being skilled in a suite of materials characterization techniques. Many of us have had the experience of needing to perform a measurement that cannot be done with standard equipment, so we learn to build or modify our own. Likewise, available analysis packages are often not suited to our purposes, so we must develop new analysis tools. Such a broad knowledge basis necessitates a broad training. Many physical chemists learn by working closely with mentors, such as PhD advisors, postdocs, and senior graduate students, who teach and set an example over the course of their training. However, intensive one-on-one training is inherently difficult to provide to all researchers in the field as it takes a great deal of time from a relatively small pool of experts. This causes issues in terms of diversity, equity, and inclusion (DEI), as socioeconomic and national barriers exist to many who would benefit from opportunities to train alongside experts.^1^ As we think toward the future, increasing opportunities for training and education in physical chemistry should be a key focus for the field.

As we look forward ∼30 years to the future of physical chemistry in 2050, it is useful to recall the radical changes of the last 30 years. Before the advent of personal computing, data was laboriously gathered on instruments which produced analogue printouts of readings. Instruments that were connected to computers required floppy disks for data transfer. Consequently, digital analysis was only viable with data sets fitting onto these drives using only procedures that did not demand significant processing. As a result of these limitations, as well as the preponderance of relatively unapproachable coding languages of the time (FORTRAN, C), data analysis/fitting was often done by hand. Scientific instruments of the time also reflected these constraints: high degrees of automation, multichannel detection, and digital preprocessing were rare. Science communication has also radically changed: fax machines, land lines, and mainframe computers were the standard 30 years ago. Preparing manuscripts involved typists, hand-drawn figures tediously and meticulously prepared by draftsmen, and submission of papers was done through the mail and could take many months to proceed.^2^ Although there are many domains, legacy instruments, procedures, and analysis modes persist, generally speaking, computers, approachable coding languages and software packages, graphical user interfaces, and digital connectivity through the internet have changed how we gather, analyze, and disseminate scientific information.

What then can we expect to see in 2050 that will change physical chemistry? And how will these changes impact growing inequities in training and mentoring the next generation of physical chemists? Just as digitization and connectivity were at the cusp of wider penetration into science in the 90s, today there are technologies that are changing how science is done broadly, with specific impacts for physical chemistry. In terms of approaches to science, a continued drive toward open-sourcing and freely available distribution of hardware schematics, codes for hardware automation and data analysis, and raw data sharing is beginning to rapidly take hold. Open sourcing scientific data acquisition and dissemination yields increased transparency, allows other scientists to utilize data sets, and can lower barriers for researchers who are entering a new field or learning a new technique.^3−5^ For example, open hardware holds promise for making both advanced (in situ, time-resolved, and others) and basic instruments cheaper and more available. Open data analyses and codes for automating workflows, disseminated on cloud-based software development platforms such as GitHub, allow for increased transparency in how scientists have reached conclusions in their data, and allow for others to build off of these tools. Open educational resources enable more researchers to learn the fundamentals of a field by accessing a plethora of videos, lecture notes, and other tools available on the Internet.

Recent technological advances are also beginning to make an impact on education in physical chemistry. Artificial intelligence (AI), in the form of large language models (LLMs) like OpenAI’s ChatGPT, Google’s Gemini (formerly Bard), as well as specialist coding companions such as Github’s Copilot, are already beginning to disrupt and change how different aspects of chemistry are done. Chemical educators are already experimenting with using these tools to hone students’ scientific writing skills.^6^ Educators are also grappling with the ability of AI tools to solve relatively difficult college-level calculus and physics problems.^7^ Additionally, many aspects of chemical science research from literature searching, coding, data analysis, and robotic operation are already being accelerated using AI tools.^8,9^ There are, of course, concerns about the use of AI tools like ChatGPT in education,^10,11^ stemming from the propensity of these LLMs to “hallucinate”; confidently producing responses that sound correct, but are factually incorrect. Already publishers like the American Chemical Society^12^ and The Royal Society^13^ have laid out standards for reporting the use of LLMs in manuscript preparation, emphasizing proof-reading AI generated output. However, we must prepare for the likelihood that integration of AI tools will become as ubiquitous as the integration of the internet is today. We as physical chemists should therefore anticipate and respond to this changing technology. As early career research fellows working on the open-sourcing of technical education, data analysis, and instrument development, and AI, we are particularly interested in ensuring that technological advancements do not lead to the use of technical resources without understanding how they work (e.g., treating a tool as a “black box” no appreciation of potential downsides and limitations). How then can we ensure that rigorous science is still done in a future where technological tools do a larger and larger share of knowledge-based work?

**Figure 1:**
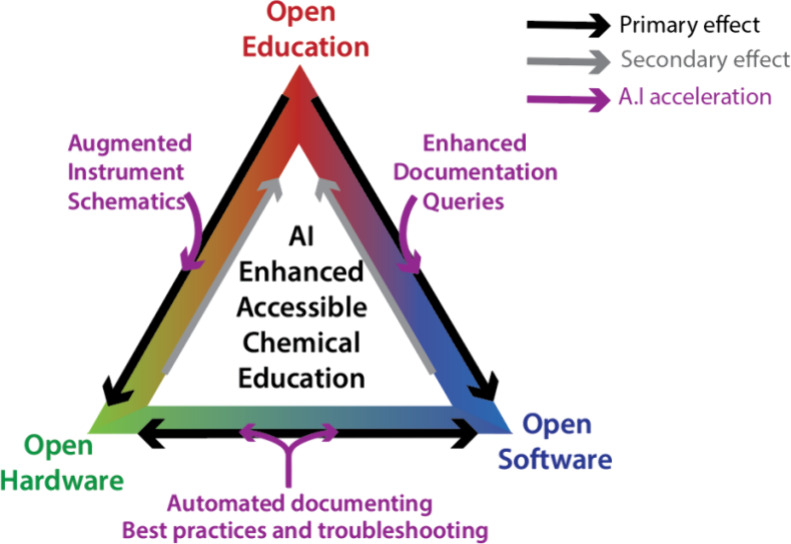
Open education generates a user and developer base for open-source chemical hardware and software (“primary effect”). This community enhances practical education through the provision of clearly documented and easy to use instrumentation and software packages (“Secondary effect”). A rapid expansion in the scale and penetration of this approach is facilitated by AI (“AI acceleration) which speeds development and enhances education.

In this Perspective, we provide a vision of a future for physical chemistry that incorporates wide adoption of open and freely available educational resources, data analysis tools, and hardware set-ups for data acquisition, specifically taking into account anticipated advances in technologies such as AI open access to knowledge and transparent sharing of technical resources will democratize the process of learning physical chemistry, where everything from fundamentals to complex data analyses and novel instrumentation are made readily accessible through open sharing of resources. The AI revolution represents a potential step change in the speed at which open resources can be developed and documented, where the uptake of these new resources by users will be accelerated through integrated educational tools. Such free sharing of the intricacies of data collection and analysis will improve education not only in the classroom setting but also within research laboratories that are entering a new subfield. This inter-relation between open education, data analysis, and hardware is represented schematically in Figure 1.

Although a future of widely available resources comes with risks of misuse, we also foresee that technology can play a role in ensuring that future physical chemists will be able to use and understand these tools. By encouraging expert practitioners to create educational resources such as tutorial videos, demonstration instruments, perspective/methods articles, data analysis scripts, detailed package documentation, and other indexable media, the impact of each expert will be broadened and bolstered by technological tools that are trained on a plethora of physical chemistry expertise.

## Open Education

Educating future physical chemists is paramount to the prosperity of the field, especially in light of an ever-changing technological landscape. Thirty years ago, physical chemists were witnessing advances in computing and connectivity. It would have been difficult to imagine the impact these technological advancements would have in all aspects of chemistry, from information acquisition, data acquisition, analysis, to scientific publishing. In 30 years from now, we should expect similar technological advances to change how physical chemistry (and chemistry broadly) is done.

One thing, however, that has remained consistent over the years is that physical chemistry expertise is often acquired under intensive one-on-one training and mentorship from experienced colleagues, such as research advisors, postdocs, and senior graduate students.^14^ The subtleties of designing experiments with proper controls, knowing what techniques to employ to answer a particular question, and how to parse data from noise/spurious results are crucial in physical chemistry training. While it is possible to pick up these skills, there is a fundamental problem: one-on-one mentorship in research groups that have the proper personnel and expertise, almost by definition, cannot scale. Research groups can only support a finite number of trainees, and mentorship takes a great deal of time and resources. Additionally, there are many barriers to accessing such training, including location, language, and cost constraints, which restricts opportunities and decreases equity.^15^ Compound this with a global increase in the number of researchers (up 13.7% from 2014 to 2018^16^), an ever-increasing number of publications (up >5% annual growth rate^17^) and the issue of being able to train the next generation of physical chemists in an equitable manner becomes apparent. Thinking toward the future, how can we address these looming issues in physical chemistry education for all researchers while keeping education rigorous?

There are many free online resources for understanding foundational chemistry concepts (e.g., ChemLibreTexts, Khan Academy, etc.). Additionally, more formalized courses created by Universities such as massive open online courses (MOOCs), which offer free full courses consisting of online video- and text-based materials, are readily available for one to learn physical chemistry.^18,19^ Such resources are part of a larger movement toward Open Education, which has been defined by entities such as SPARC as “...resources, tools, and practices that are free of legal, financial and technical barriers and can be fully used, shared and adapted in the digital environment,”^20^ with the broader goal being democratizing education. However, when it comes to more advanced physical chemistry techniques, analyses, and instrumentation encountered in scientific research, fewer resources are available. High-level expertise is instead more likely distributed across many papers in the literature, and typically written in highly technical language geared toward topic experts. To mitigate this, we as domain experts need to make efforts to produce educational resources to clearly explain our technical niches. Currently this can take the form of experts providing lectures/webinars that are made freely available on special topics. Some excellent examples include webinar series such as the Physical Inorganic Tutorials,^21^ Electrochemistry Colloquium (https://www.electrochemicalcolloquium.com/), or online resources for producing and curating pedagogical content such as the Virtual Inorganic Pedagogical Electronic Resource (VIPEr). Additionally, posting lecture videos from university-level coursework (introductory or otherwise) is also particularly valuable, although this may pose challenges in terms of rights/ownership. Contributing to and editing Wikipedia articles on advanced physical chemistry topics can also improve the accessibility of knowledge in the field, especially considering the widespread use of Wikipedia articles by the recent generation of students and researchers.^22^

It is also important that domain experts provide (peer-reviewed) perspectives to newcomers in the field. A newcomer scouring the literature will often find it difficult and time-consuming to get a sense of what common mistakes one should avoid when doing research, especially when many incorrect approaches are perpetuated in published literature. Editorials that highlight pitfalls in research^23−27^ or those that are a call-to-action to avoid unscientific research that dazzles but lacks substance^28−30^ are especially useful for newcomers (e.g., see collection from ACS Energy Letters on pitfalls to avoid in catalysis^23^). Similarly, methods/protocols papers that lay out the basics of how to get started with a new technique or approach are particularly important (1) for making new areas of research accessible,^31^ but also (2) for standardizing approaches^32−34^ to avoid proliferation of poorly executed work and misinterpretation or overextrapolation of results. The utility of such publications is evidenced by the sheer amount of downloads these publications have received. Notable examples include one that focuses on fabricating halide perovskite solar cells^35^ (31,836 article views), performing cyclic voltammetry measurements^36^ (877,642 article views), or understanding the basics of X-ray diffraction for nanomaterial research^37^ (144,969 article views; all views as of 2023/12/19).

Today such methods/protocols resources can be readily enhanced by adding photos and videos, likely taken on the average smart phone, and uploaded to a Supporting Information section to provide a first-hand account of how to use a new technique. In the future, virtual/augmented reality (VR/AR) videos will be even easier and more accessible to produce and can easily enhance these resources. Imagine being able to walk through step-by-step, in a 3D environment, and see each fundamental step of setting up and performing a physical chemistry procedure (synthesis, measurement, etc.). Producing such methods/protocol resources is important for near-term dissemination of knowledge, but we must also consider that such resources will undoubtedly be used to train future generative AI models, which will enhance future educational resources. It is important to note that tutorial articles such as those mentioned above are better judged based on their views/downloads, as new researchers will likely utilize these resources to help in their experiments but may not necessarily cite it. This is one of many deficiencies to how we evaluate and reward researchers through citation indexes. We as physical chemists must advocate our institutions to instead take a more holistic approach when evaluating academic output, as many important resources (videos, lectures, GitHub code, etc.) are not necessarily captured in a simple metric such as an H-index, where emphasis is instead placed on citations.

The above-mentioned examples show what resources physical chemists can produce today that will benefit physical chemistry education. However, thinking toward the future of physical chemistry, what can we expect to change, and how can we contribute to improving education? While AI is currently adept at analyzing and producing text, the readability of images and video content is still in its nascent stage.^38^ However, projecting 30 years into the future, one can anticipate that there will be greater fidelity in image and video analysis. Such AI image analysis could greatly enhance expert-derived educational resources that are video-based, allowing for more interactive media to influence education. For example, in 30 years, we might be able to watch a recorded lecture, pause the video, and utilize an AI-based tool to ask questions about what we are being taught on the screen. If something was only briefly mentioned in a piece of educational content, a well-trained AI tool could be used to expound upon what might have been a single sentence provided in a piece of educational content. Already there are efforts to weave AI Chatbots into introductory chemistry courses,^39^ which increases student engagement and allows for real-time feedback. This could be especially helpful in flipped classroom environments, where students watch video lectures before coming into lab/class. Studies of flipped classroom approaches have shown that nearly a third of students watch prerecorded videos more than once, indicating that they did not fully understand the material^40^ - a chatbot that can answer questions in real-time could enhance the learning process. However, current implementations of AI in online coursework are somewhat limited and are only trained on that specific online course.^41^ Future chatbots will likely be adept at engaging with content generated across the wider Internet, where a larger training data set is used.

We anticipate that both developments in AI, along with virtual/augmented reality (VR/AR), will play a critical role in future physical chemistry education. In the future, one can also envision that a physical chemist could, for example, easily and cheaply produce a 3D or VR/AR video walkthrough which explains the basics of a home-built physical chemistry instrument setup. Imagine being able to pause a video walkthrough of a custom laser spectroscopy setup in a 3D environment and asking a chatbot what a certain piece of equipment in the frame does–let us say a lock-in amplifier. Future AI tools will likely be able to carry on conversations and supplement video-based educational resources beyond what was originally provided by the expert/mentor who created them. Virtual reality approaches have been explored in physical chemistry education, such as in molecular dynamics^42,43^ and elementary reaction kinetics,^44^ with students reporting improved educational outcomes. In the future, the ability to produce VR/AR videos as easily as pulling out one’s phone and recording will allow for more immersion and greater depth of communication, while democratizing the creation of high-quality educational resources. Supplementing these resources with AI chatbots that can process and analyze video will allow for a given piece of educational content to have a greater degree of interaction and utility than the original creator could provide.

**Figure 2:**
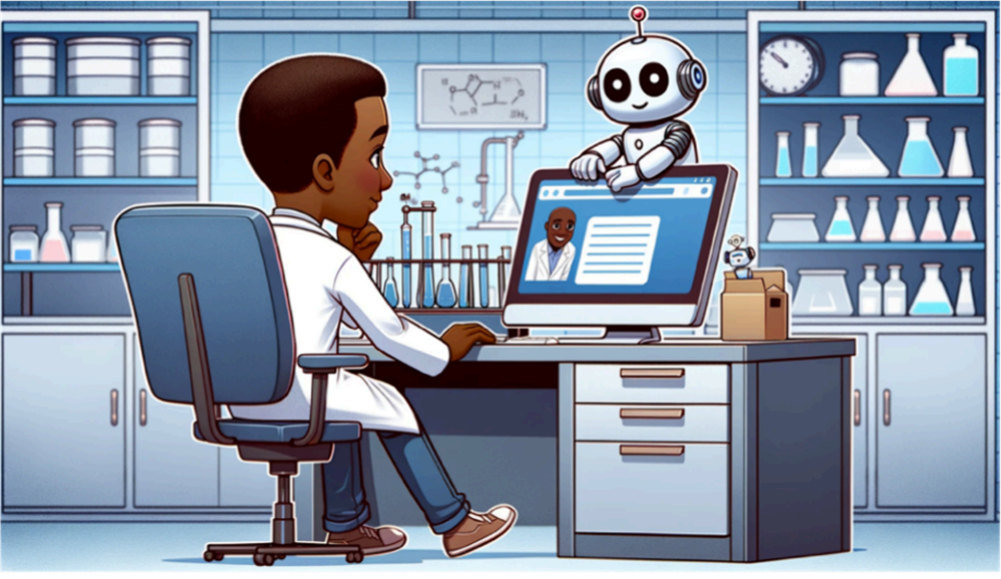
Illustration of a physical chemist of the future, learning using educational resources generated by experts, enhanced by Al chatbots that can act as reliable tutors. Image generated by DALLE-3/ChatGPT-4.

Although such a future might seem somewhat far-fetched, there are already indications tools are advancing toward this goal. For example, OpenAI, creators of ChatGPT, have recently announced that users can create custom GPTs that are tailored toward specific tasks, and trained on specific inputs.^45^ One can easily imagine that future LLMs can be trained not only in the fundamentals of chemistry, physics, biology, etc., but also be tailored to teaching specialized topics. This task-specific training is akin to GitHub’s Copilot AI tool, which is trained on many gigabytes of open-source scripts and troubleshooting logs in dozens of programming languages to be able to autocomplete code and suggest code blocks based on comments.^46^ Future educational tools will presumably be trained on an entire body of textbooks, instructional videos, and scientific publications/preprints. In other words, with enough educational resources generated by experts in a physical chemistry subfield, a large degree of high quality, reliable, and accessible knowledge will be made freely available to allow for AI tutors that can have conversations with a learner (see Figure 2 for an illustration). By 2050, we can also assume that such AI tools will be proficient in “speaking” in most languages (i.e., reading and responding to prompts in different languages), which will further democratize learning.^47,48^ Already chemical educators have begun leveraging curated chatbots to help students hone their scientific writing skills^6^ - a trend of AI integration that will only continue. Similar to how free and open online resources such as Khan Academy and MOOCs are able to close the learning gap and bring students up-to-speed for university coursework,^18,49^ we expect expert-generated educational content enhanced by AI tools to serve as an approach to lower barriers for researchers at higher levels of scientific research.

As we think to the future of open education, we should anticipate that technological advancements will play a key role. AI tools that can enhance educational resources will likely be the norm, with learners being able to interact with a chatbot-like tool to ask questions and clarify content. This will not replace one-on-one training or mentorship; instead, it will allow mentors and experts in physical chemistry to have their expertise extend far beyond their own laboratories and their own professional circle.

## Open Software/Analysis

Scientific software now touches every corner of the physical sciences and serves many purposes. Free software (for which, in the present Perspective, we will use “open software” as a synonym), is defined by the Free Software Foundation^50^ by the following four conditions, which each bring advantageous in science, education, and science education:

- The freedom to use the program in any way, intended or unintended by the author(s). This enables innovation and experimentation by both scientists and students.
- The freedom to examine and modify the source code. This facilitates transparency, allowing scientists and students to “look under the hood” both for scrutiny and for inspiration.
- The freedom to redistribute copies. This removes cost barriers to scientists and students working with the same software, enabling standardization and facilitating mass learning.
- The freedom to redistribute modified copies. This invites everyone with time and interest into the development of scientific software. Not only does this facilitate its continued improvement, it can also be immensely inspiring for students to see their improvement incorporated in software.

“Open data analysis” is the sharing of data analysis procedures according to the same four criteria, for example as a repository of scripts which show how to produce article figures from starting data accompanying a publication. Open data analysis is almost always *programmatic* data analysis, because a script in a programming language is the most precise way to convey which steps were taken. Open data analysis is a growing and positive trend, which we expect to be widespread if not universal in physical chemistry long before 2050.

Strong arguments have long been made across disciplines that scientific publications should include or make available and point to the source code needed to do the calculations supporting the paper’s findings.^51−55^ Calls for open data analysis strengthened as the challenges in reproducibility and replicability across the sciences became clear.^53^ Scientists and students need to be able to see how data analysis was done to better follow and reproduce scientific results and iterate on those results. When data analysis is done with proprietary software, it creates a barrier for all students and scientists who do not have access to the same software. Additionally, descriptions of data analysis methods published in articles are subject to a trade-off between conciseness and completeness, so in lieu of available code it is often impossible to follow exactly how data analysis workflow was done without contacting the scientists who performed the work.^55^ For these reasons, the current lack of open data analysis contributes to the reproducibility challenges that physical chemistry faces.

Barriers include reliance on proprietary software or nonprogrammatic data analysis, lack of incentives for sharing code, and reluctance to share imperfect code. All of these barriers can decrease with improved education in programming, open-source development workflows, and data science. While sharing of data is increasingly required, and standards of data availability have been established such as the FAIR principles (that research data should be Findable, Accessible, Interoperable, and Reusable) requirements and standards of code sharing are inconsistent and ambiguous.^56^ A number of organizations across disciplines are helping by providing educational resources and tools to assist with open scientific data analysis including the Center for Open Science^57^ and the Framework for Open and Reproducible Research Training.^58^

Within the field of physical chemistry, at present, computational chemists have made the most progress on this (see e.g. Rossmeisl Group’s KatlaDB,^59^ and Aaron Walsh’s Materials Design Group codes^60^) while experimentalists in the physical chemical sciences have often lagged behind. The widespread adoption of open data analysis is challenging, partly because many scientists are turned away by the needed programming skill, and partly because it is both time-consuming and not necessarily recognized for one’s scientific career. The Journal of Open Source Software^61^ helps incentivize code sharing by facilitating peer-reviewed publication of code packages that can then get cited.

Open data analysis is facilitated by new tools that are making it easier to learn how to program and share code in readable formats. Countless free online tutorials exist for popular open-source programming languages like python. For example, the advent of Jupyter notebooks in 2014^62^ enabled the mixing of code and rich-formatted text fields for attractive tutorials. Since 2007,^63^ Github has made it easy to work together on data analysis scripts within a team as an initially private repository, and then open it to the public when the data is published. Today, new AI tools that can assist users write their own code, such as Github Copilot, are further lowering barriers to allow more physical chemists to develop their own code.

Open scientific software also facilitates open data analysis by packaging and standardizing often-used data analysis operations. Broad-scope examples in physical chemistry include the atomic simulation environment (ase)^64^ and the in situ experimental data tool (ixdat).^65^ Scientific code packages for narrower use cases abound, as seen, for example, in the Journal for Open Source Software. However, one pitfall can arise when too many packages are produced, with many groups unnecessarily reinventing the wheel. We believe there should be increased awareness in how to find whether there is an existing package to build on rather than starting from scratch, and we encourage developers to keep in mind that many valuable additions to the available scientific codes are best made in collaboration with existing free software projects.

Another key to progress in physical chemistry is open databases. Examples include the Materials Project, Crystallography Open Database, and many others.^66,67^ These databases are queried and expanded via open software packages. Chemical innovation is largely expected to be accelerated by Machine Learning (ML), with open databases as a key enabler.^68^ These can only work with large quantities of standardized and high-quality data. Working out the details and collecting the data has to be a community effort that scientists and students can feel rewarded for contributing to. We hope to see more databases of experimental data that are easy to contribute to including ones of UV–vis spectra, mass spectrometry calibrations, battery charging curves, and (electro-)catalytic activity, as just a few examples. As these databases grow, ML will improve as a tool for material design.

By 2050, we envision that essentially all software used in scientific education and research will be free software, as defined above. Scientific software and data analysis will be consistently shared following clear guidelines aligned with FAIR principles. Programming will be taught as an essential skill on par with mathematics and scientific language, and visualization tools will make it possible to view and navigate the design of complex programs by interpreting the structure from the source code. Students and scientists will be at complete ease typing one line of code to plot their data and another to coplot it with the most trustworthy data from the literature, fetched automatically from community-maintained databases. Scientific publication will no longer be a confusing cacophony of unreliable record-breaking claims, but a collaborative community project with relations between each discovery linked to the raw data behind it and visually mapped to its broader context.

## Open Hardware

Over time, the requirements for publishing have changed. Thirty years ago, many spectroscopic or material characterization techniques were primarily operated by specialized groups (e.g., transmission electron microscopy). Since then, commercial instruments have been refined and simplified to become *turn-key solutions* accessible to a wide user base. Many universities today have material characterization facilities, which are often filled with *ex situ* X-ray-based techniques (XRD, XPS, etc.) and spectroscopic set-ups (FTIR, Raman, UV–vis, photoluminescence (PL)), among others. Consequently, a suite of characterization methods are a common prerequisite to publication. However, *in situ* and *operando* techniques, which probe structure and function during operation, typically remain the domain of specialist groups and synchrotron facilities. The abundance of turn-key *ex situ* characterization and the relative scarcity of accessible technical education in these techniques has created educational challenges and opportunities. Even the simplest measurement has pitfalls: a classic example being the diffraction grating in a UV–vis or PL system, which passes integer multiples and fractions of a selected wavelengths on detectors, which also can easily be saturated. Thus, the expansion of turn-key *ex situ* characterization has led to an uptick in artifact ridden measurements and misinterpreted data, as evidenced by the growth of articles seeking to correct these issues (see for example this resource for PL measurements^69^ and this for absorbance^70^

The problem of misusing instruments is pervasive and would benefit from a more general approach to technical education. Meeting challenges in technical education and the development high-quality, low-cost instruments are key drivers of the open-source scientific hardware (OScH) movement.^71^ The term Open Source Hardware refers to “*tangible artifacts—machines, devices, or other physical things—whose design has been released to the public in such a way that anyone can make, modify, distribute, and use those things*.”^72^ The goals of this movement are to make scientific hardware more socioeconomically accessible, replicable, and performant.^73^ Reflecting the need to develop across the spectrum from high cost/low accessibility to low cost/high accessibility, the OScH movement aims for “*implementation of OScH projects across highly unequal contexts with respect to industrial and infrastructural access, [and] socioeconomic divides”*.^71,73^ We envisage this approach to have significant utility in filling the technical knowledge gap in physical chemistry by standardizing and optimizing demonstrator instruments as well as providing cutting edge instruments at minimal cost to institutions in areas that are more socioeconomically strained. Open source and freely available hardware schematic documentation holds the potential to educate users from the undergraduate level up. The benefits can broadly be categorized into the following:

**(1) Demonstrator systems for basic technical education and accessibility. High-quality instrumentation for advanced training.** Technical education is impossible without proper equipment. However, such equipment is inaccessible to many. This problem can be addressed by supplying simple and low-cost demonstrator systems to be built and operated by students in teaching laboratories. By building and operating instruments, a hands-on approach to best practice can reinforce the understanding gained through open education, thus creating a holistic approach to learning. To illustrate this point, we return to the case study of a UV–vis spectrometer. At the time of writing, an Agilent Cary 60 “dual beam” UV–vis spectrometer can be purchased for £8130 (after tax)—an inaccessible sum to many laboratories and institutes. A simple dual beam UV–vis setup can be constructed for under £100, using plastic gratings, bootstrapping a smart phone camera for a detector using an app in order measure intensity,^74^ while the key components for a spectrometer with equivalent sensitivity and resolution to the Cary 60 can be purchased for under £2500 using a standard Czerny–Turner configuration using parts from well-known optics supplies.^75^ To the best of our knowledge, many open spectrometers focused on low cost and accessibility rather than resolution/sensitivy.^74,76^ However, invaluable advanced skills are obtained in the design of more advanced instruments. These skills in turn facilitate the design of powerful specialist instruments, such as *operando* spectrometers. Currently, the barrier for the uptake of advanced education in instrumentation is the accessibility of educational resources, high quality schematics, and parts lists. In the future, we anticipate fully integrated educational projects aimed at the production of the entire spectrum of instruments, ranging from demonstrators to world class, lab grade instruments for as low a cost as possible.

**(2) Accessibility of and education in specialist techniques.** The confinement of *operando* and time-resolved techniques to specialist groups and institutes can be a result of the need for intrinsically inaccessible installations or components, such as is the case for synchrotron techniques and the lasers used in ultrafast spectroscopies. However, many techniques have high barriers to entry because of the inaccessibility of advanced technical education. The jump in technical skill between the ability to design, build and automate a relatively simple Czerny–Turner monochromator/spectrograph or a Michelson interferometer, compared to the skills needed for many specialist *operando* and time-resolved techniques (e.g., transient absorption, time-resolved single photon counting, spectroelectrochemistry, and time- and potential-resolved Raman and surface enhanced FTIR), is smaller than one might imagine. However, such expertise typically resides within specialist research groups and is passed on by one-on-one mentoring using home-built systems. These systems are often the result of years of ad-hoc development. Consequently, there is typically a steep learning curve to understanding how these systems work, requiring the mentee to learn to distinguish esoteric and legacy operating procedures from fundamental measurement principles. In this process a mentee will experience the pitfalls of the measurement, learn to distinguish signal from noise, troubleshoot problems, and use powerful analytical procedures.

**(3) Robust knowledge transfer.** The chain of mentoring is vulnerable to loss of expertise, as well as geographic/sociopolitical inequalities and systemic biases. The COVID-19 pandemic has greatly exacerbated the common issue of a postdoc/grad student leaving with all the knowledge of a setup, leading to the abandonment of instruments. Geographic/sociopolitical inequalities, combined with the abstruse nature of many home-built setups, means that even if financial resources are secured for the commission of a new instrument, additional funds must be obtained for a student or researcher to travel to another lab to learn a new technique/instrument setup. These problems can and should be addressed by OScH. In the creation of optimized and standardized instrument schematics, performance can be increased and costs decreased. Local commission of instruments will made possible by logical design, clear documentation, and software support. This will be supported and reinforced by open educational resources such as video lectures, workshops and inductions, each supplemented by AI-based tools for answering a user’s questions.

A barrier to the advancing these goals is the reward systems that are currently in place. For example, besides just tracking paper citations in academic metrics, one can envision a future where, for example, engagement with free resources on GitHub is tracked as an academic metric, hopefully encouraging the production of free hardware/software tools. In the long term is crucial to improve reignition metrics, funding, and increase the number of prizes and awards which support and legitimate OScH, such as the Open Hardware Creators in Academia prize, which aims to recognize leaders in scientific open hardware and provides financial support to grow new collaborations and projects in in OScH, we anticipate a future for physical chemistry were advanced technique research groups also support high quality instrumentation, software and documentation, because researchers are recognized and rewarded for this work. This trend of free hardware resources is beginning to emerge in other disciplines, for example the Holden Lab has produced and supports the LifeHack microscope platform:^77^ a flexible system for resolving single molecule fluorescence dynamics in microbes. The system is designed to reduce setup barriers while maintaining the flexibility to adapt the microscopy setup for other purposes. Simple demonstrator systems also expose researchers to concepts at the frontiers of physical chemistry. For example, autonomous laboratories driven by Bayesian search methods and machine learning are currently the subject of significant debate and interest,^78^ as searching for a better catalyst or a more desirable material is a common goal in chemistry. A wider appreciation of Bayesian hypothesis testing and experimentation would thus be beneficial for many in the community. However, the experimental context in which this is most commonly encountered, autonomous systems and robotics are beyond the experience and budget of the vast majority of physical chemists. Sparks and Baird have developed a simple device that acts as a demonstrator of an autonomous lab that can be built for under $100.^79,80^ The aim of this simple autonomous color matching device is to introduce the user to more efficient search methods for finding an optima in a high dimensional space of properties. OScH is the least developed and most contingent element of Figure 1, as OScH developers must first be fluent in both the science and software. The need for core physical chemistry skills, familiarity with instrumentation, hardware and software leads to a smaller in developer base. Further, organizing bodies, such as the Gathering for Open Science Hardware (GOSH),^73^ have only formed relatively recently–reflecting the early stage of development of OScH in comparison to Open Software or Open Education. We anticipate the AI revolution empowering hardware developers with tools to automate the production of interactive training in hardware, for example by producing interactive schematics. Our hope is that AI tools augment open hardware schematics and documentation, allowing users to ask questions of AI chatbots to clarify the role and operation of hardware components. However, OScH will still require a significant commitment of time and resources. Institutional recognition of the benefits of OScH to wider community is therefore critical to drive forward technical education in physical chemistry.

## Concluding Remarks and Call to Action

Technological advancements have drastically changed chemistry in the last 30 years. We anticipate that both technological advancements and a push toward open-sourcing will be at the heart of how physical chemistry is done in 2050. Key to the success of future physical chemists and their trainees will be proper education in the fundamental “nuts and bolts” of many of the open analysis, hardware, and software tools of the future function. This will require the experts and contributors of these projects to take time to document their tools. Documentation will likely be aided by AI tools, in order for them to be better utilized by physical chemists. Open source projects outside of chemistry provide a template, and can serve as both a source of inspiration or even be directly expanded to serve physical chemistry community. Further, we anticipate that AI tools will further be able to act as assistants to help users of these tools understand the fundamental underpinnings of why the tool works, based on a massive body of educational resources that have been made available.

Generating measurable and meaningful change in a relatively short time frame, however, is contingent on accelerating change at institutional and grassroots levels. Funders and universities must reform their reward structures, while researchers must drive interest and advancement though the development of open-source projects. The burden of creating, maintaining, organizing and promoting open-source resources is too often unrecognized by decision makers in spite of the large returns on investment that such work represents. For example, the digital economy is heavily reliant on the unpaid labor of developers of open-source code and protocols for even the most basic operations.^81,82^ In academia, OScH has been estimated to produce returns on investment of 10–100-fold, due to the large decreases in equipment cost in comparison to even simple commercial counterparts being multiplied over multiple grants.^83,84^ Highlighting the clear and accruing returns of open source work, the benefits of recognition and funding of this to decision makers setting policy at government, funder, and university levels are therefore crucial prerequisites to our vision of open source learning. Some evidence exists that this change is underway at the funder level: the National Science Foundation (NSF) encourages researchers to budget costs associated with open-source publication and has recently announced a $26 M grant focused on supporting the organizers of open-source ecosystems for the creation of new technology solutions. However, a lack of systemic recognition remains a key roadblock to the development of new open-source education projects. Over the next decade, it will be crucial that this work gains more recognition in tenure and promotion applications, direct encouragement in grant applications, and recognition as an important service to the community. We also encourage the development and sponsorship of new prizes and awards for exceptional efforts in this field, such as the Open Hardware Creators in Academia prize, which aims to recognize leaders in scientific open hardware and provides financial support to grow new collaborations and projects in in OScH.^78−81^

Further, the authors would like to stress that one-on-one mentorship will always be a key component of physical chemistry training and education; neither open education nor access to analysis/hardware tools nor new AI technologies will change this. Rather, future technological tools and open access approaches should extend the reach and increase the efficacy of expert mentors. who invest their insight and knowledge in freely available and accessible formats. In this way, more physical chemists can benefit from their mentorship, technical expertise, and perspective. However, to realize such a future for physical chemistry there are steps that we must take today. The following are actions that the authors encourage physical chemists of today to consider, in order to realize a future of democratized and open access to data analysis tools, hardware tools, and education in physical chemistry:

*Open Education*: in the short- and medium-term, physical chemists should contribute to online webinars, make tutorial videos (either themselves or as an opportunity to guide trainees to producing pedagogical content),^85^ and provide their perspective/warn of pitfalls in their research area using freely available formats such as editorials. Such resources have clear short-term benefits in education. In the longer term as technologies advance these resources can be augmented and enhanced through AI tools.

*Open Data Analysis*: in the short- and medium-term, physical chemists should accelerate the development and use of programmatic data analysis, open-source scientific software, and open databases. Successful software and databases for open science advance the case for the long-term goal of lasting funding independent of short-term research project grants to hire professional maintainers.

*Open Hardware*: in the medium-term, we encourage the development of metrics and procedures that will lead to increased institutional recognition of OScH and reward development. We suggest that developing a long-term institutional awareness of the pedogeological utility of OScH can be achieved through an increased engagement with demonstrator systems in teaching laboratories, which can be implemented relatively quickly. We encourage funders to recognize the wider benefits of the open development of cutting-edge instruments and encourage the development of OScH in dedicated in work packages. We also encourage the use of new AI tools to expedite slow documentation and troubleshooting procedures.

In summary, current practices in chemical education focused around one-on-one mentorship are inherently limited in terms of scalability, and do not address global challenges of inequality. We envisage a future where students and researchers across the world benefit from resources currently only accessible through direct mentoring. We hope that, by 2050, expansion of open scientific education, supported by carefully crafted interactive educational resources. will equip students with the grounding needed to excel. Interaction with simple pedogeological as well as cutting edge instruments will equip students with a detailed appreciation of the techniques at the core of physical chemistry. Crucially, education in practical subjects, such as physical chemistry, will be supported by powerful open-source analysis packages and instrumentation, allowing students across the world hands-on experience with designing and performing cutting edge experiments and understanding their results with powerful analytical tools.
